# Analyses of Human Milk

**Published:** 1915-10

**Authors:** 


**Analyses of Human Milk.** L. Emmett Holt, Med. Rec., Sept. 18, 1915, gives the following as averages of 29 individual specimens and 6 composite specimens for 24 hours:

| | Fat. | Sugar. | Protein. | Ash. |
|---|---|---|---|---|
| Colostrum (5 cases) ........ | 2.83 | 7.59 | 2.25 | 0.3077 |
| Transitional (6) ........... | 4.37 | 7.44 | 1.56 | 0.2407 |
| Mature (17) .............. | 3.26 | 7.50 | 1.15 | 0.2062 |
| Late (7) .................. | 2.96 | 7.45 | 1.02 | 0.2080 |

The average composition of the ash is as follows:

| | CaO | MgO | P2O5 | Na2O | K2O | Cl |
|---|---|---|---|---|---|---|
| Woman's ............. | 23.2 | 3.7 | 16,5 | 8. | 28.2 | 16.6 |
| Cow's ................ | 21.8 | 2.9 | 28.9 | 7.2 | 26.5 | 13. |

Iron in human milk amounts to about 1.7 milligram per liter. Note—The infant is protected against anaemia by a store of iron in the liver and spleen. This protection does not exist to any degree in the adult, hence the danger of prolonged milk diet. Ed.

